# Integrating Anatomical, Molecular and Clinical Risk Factors in Gastrointestinal Stromal Tumor of the Stomach

**DOI:** 10.1245/s10434-021-09605-8

**Published:** 2021-03-02

**Authors:** Toto Hølmebakk, Anne Marit Wiedswang, Leonardo A. Meza-Zepeda, Ivar Hompland, Ingvild V. K. Lobmaier, Jeanne-Marie Berner, Stephan Stoldt, Kjetil Boye

**Affiliations:** 1 Department of Abdominal and Pediatric Surgery, Oslo University Hospital, The Norwegian Radium Hospital, Nydalen, Oslo, Norway; 2 Department of Radiology, Oslo University Hospital, The Norwegian Radium Hospital, Oslo, Norway; 3 Department of Tumor Biology, Institute for Cancer Research, Oslo University Hospital, The Norwegian Radium Hospital, Oslo, Norway; 4 Genomics Core Facility, Department of Core Facilities, Institute for Cancer Research, Oslo University Hospital, The Norwegian Radium Hospital, Oslo, Norway; 5 Department of Oncology, Oslo University Hospital, The Norwegian Radium Hospital, Oslo, Norway; 6 Department of Pathology, Oslo University Hospital, The Norwegian Radium Hospital, Oslo, Norway

## Abstract

**Background:**

Adjuvant imatinib for 3 years is recommended to patients with high-risk gastrointestinal stromal tumor (GIST). Risk stratification is inaccurate, and risk assessments are further complicated by the increased use of neoadjuvant treatment. Anatomical criteria for prognostication have not been investigated.

**Methods:**

Clinical, molecular, and anatomical variables were retrospectively studied in a population-based cohort of 295 patients with gastric GIST resected between 2000 and 2018. Gastric subsite was divided into the upper, middle, and lower thirds. Growth pattern was classified as luminal, exophytic, or transmural based on imaging and surgical reports.

**Results:**

Of 113 tumors in the upper third of the stomach, 103 (91.2%) were *KIT* mutated, 7 (6.2%) were *PDGFRA* mutated, and 104 (92.0%) harbored genotypes sensitive to imatinib. Transmural tumors were strongly associated with a high mitotic index. Five-year recurrence-free survival (RFS) was 71% for patients with transmural tumors versus 96% with luminal or exophytic tumors (hazard ratio [HR] 8.45, 95% confidence interval [CI] 3.69–19.36; *p *< 0.001), and, in high-risk patients, 5-year RFS was 46% for patients with transmural tumors versus 83% with luminal or exophytic tumors (HR 4.47, 95% CI 1.71–11.66; *p *= 0.001). Among 134 patients with tumors > 5 cm, there were 29 recurrences. Only five patients with exophytic or luminal tumors had recurrent disease, of whom four had tumor rupture. Five-year RFS for patients with exophytic/luminal tumors >5 cm without rupture was 98%.

**Conclusions:**

In the upper third, over 90% of tumors were sensitive to imatinib. Patients with exophytic or luminal tumors without rupture, irrespective of size, had an excellent prognosis and may not benefit from adjuvant therapy.

**Supplementary Information:**

The online version contains supplementary material available at 10.1245/s10434-021-09605-8.

Adjuvant imatinib treatment for 3 years is recommended to patients with localized gastrointestinal stromal tumor (GIST) at increased risk of disease recurrence.^1^–^3^ These patients are identified by the established stratification systems with high sensitivity;^4^–^7^ however, specificity is low and most patients with high-risk gastric GIST will not relapse without adjuvant therapy. Neoadjuvant treatment for 6–12 months is increasingly used for locally advanced tumors and tumors in surgically difficult positions, e.g. the gastric cardia, and effectively reduces tumor volume.^8^ Upon neoadjuvant treatment, mitoses disappear in the surgical specimen, precluding accurate risk stratification, and mitotic index (MI) cannot be properly assessed in preoperative biopsies. Nevertheless, continued imatinib treatment postoperatively is recommended and is generally practiced in patients submitted to neoadjuvant therapy.^3^,^8^ Mutation analysis is considered a prerequisite for adjuvant or neoadjuvant therapy, as approximately 25% of GISTs harbor a genotype not sensitive to imatinib.^9^,^10^ However, in parts of the world, facilities for biomolecular analysis are lacking, or services may be too expensive for routine practice.^11^

Gastric GISTs carry a better prognosis than tumors of the small intestine.^10^ Smaller size and lower mitotic activity partly explain this, but gastric GISTs still have a favorable prognosis compared with non-gastric GISTs of similar size and with comparable MI.^12^ Molecular characteristics have predictive as well as prognostic implications in GIST and they differ in gastric and non-gastric tumors. Tumors wild-type for *KIT* or *platelet-derived growth factor receptor-α (KIT/PDGFRA)* and *PDGFRA* exon 18 D842V-mutated tumors are not sensitive to imatinib, and the latter is almost exclusively found in the stomach.^9^ These genotypes have a good prognosis, whereas tumors with *KIT* exon 11 deletions involving codons 557 and 558 (del557/558) are associated with an aggressive phenotype in the stomach.^9^

Mutation status is not incorporated in the staging systems, neither are anatomical features. Organ subsite is a recognized prognostic variable in gastrointestinal carcinomas,^13^,^14^ but has hardly been investigated in GIST. Characteristic of gastric GISTs is their macroscopic growth pattern: luminal, exophytic, or transmural. Whether these patterns represent similar neoplasms arising from different layers of the gastric wall or are associated with different clinical or biological properties, is so far not known.

The present study was undertaken to give a comprehensive picture of gastric GIST in a population-based series with the aim of improving prognostic accuracy, thereby making adjunctive medical treatment more specific.

## Methods

### Patients, Treatment and Follow-Up

Patients who underwent complete excision (R0/R1) of primary gastric GIST between 1 January 2000 and 31 August 2018 were identified in the sarcoma database of the Oslo University Hospital. Patients with synchronous metastases and multifocal primary GIST were excluded. Oslo University Hospital is a sarcoma center for the South-East Health Region of Norway, with a population of 2.9 million. From all hospitals and laboratories of pathology, reporting cancer cases to the Norwegian Cancer Registry is mandatory. To verify the completeness of the sarcoma database at Oslo University Hospital, this registry was contacted. The number of patients with resected, localized gastric GIST in the sarcoma database corresponded to 98% of the patients reported to the Norwegian Cancer Registry from the South-East Region.

The diagnosis of GIST was made by sarcoma pathologists according to established criteria.^15^ The administration of adjuvant imatinib and follow-up have been described previously.^16^ Recurrence was recorded if verified on biopsy or indisputable on computed tomography (CT). Recurrence-free survival (RFS) was measured from the date of surgery to recurrence. Patients were censored at the date of the latest CT, and, for patients who died from surgical complications, at the date of surgery. The study was approved by the institutional Data Protection Officer and mutation analysis using next-generation sequencing (NGS) by the Regional Committee for Medical and Health Research Ethics of South-East Norway (No. 2010/1244). Written informed consent was obtained.

### Clinical Data and Definitions

**Fig. 1:**
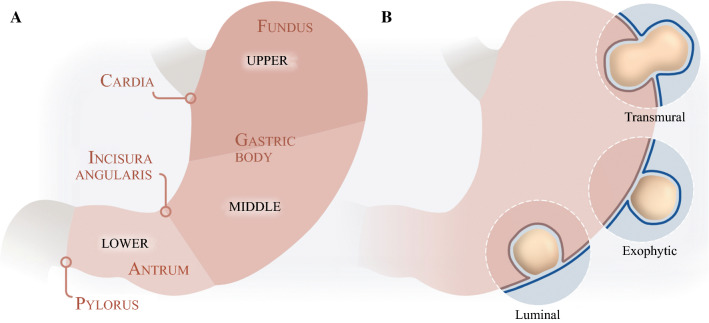
**a** The partition of the stomach into the upper, middle, and lower thirds. **b** Macroscopic growth pattern: luminal, exophytic, and transmural tumors

Prospectively collected data from the database were supplemented from medical records and additional mutation analyses were performed. Mitoses were only counted in imatinib-naïve patients, and not in biopsies. Risk of recurrence was stratified according to the modified National Institutes of Health (mNIH) criteria.^5^ For patients who received neoadjuvant imatinib treatment, only those with tumors larger than 10 cm on initial imaging or tumor rupture were stratified (high risk) (Tables 1, 2). Tumor rupture was defined according to the Oslo definition.^16^ Gastric subsite was divided into the upper, middle, and lower thirds (Fig. 1a). Macroscopic growth pattern was classified as *luminal*, not affecting the peritoneal contour of the stomach; *exophytic*, not affecting the mucosal contour; or *transmural*, affecting both the peritoneal and mucosal contours (Fig. 1b). The assessment was independently performed on CT imaging by a radiologist (AMW) and a surgeon (TH) [interobserver agreement 90.0%, *κ* = 0.85] and resolved by consensus when not in agreement. If the surgical report indicated a growth pattern different from the images, the surgical assessment was chosen. Patients with a history of mucosal ulceration (bleeding) or ulceration detected on microscopy/endoscopy were, by definition, classified with a luminal component; similarly, microscopic serosal penetration was equivalent to an exophytic component.

**Table 1:** Clinical, pathological, and biological characteristics

Total number of patients	295
Sex, males:females	149:146 (50.5:49.5)
Age at surgery, years [median (range)]	66 (14–93)
Syndromic GIST	1 (0.3)
Presentation	
Symptomatic	193 (65.4)
Bleeding/anemia	99 (33.6)
Pain/discomfort	57 (19.3)
Other	37 (12.5)
Incidental finding	102 (34.6)
At imaging/endoscopy	90 (30.5)
At surgery	12 (4.1)
R1 resection	28 (9.5)
Multivisceral resection	41 (13.9)
Tumor rupture	24 (8.1)
Gastric subsite	
Upper third	122 (41.8)
Middle third	120 (41.1)
Lower third	50 (17.1)
Borderline or unspecified	3
Macroscopic growth pattern	
Luminal	100 (34.1)
Exophytic	94 (32.1)
Transmural	99 (33.8)
Unspecified	2
Tumor size, cm [median (range)]	4.8 (0.5–34.0)
≤ 5	161 (54.6)
5.1–10.0	83 (28.1)
> 10	51 (17.3)
Mitotic index, per 50 HPF [median (range)]	2 (0–166)
0–5	221 (77.9)
6–10	25 (8.8)
> 10	38 (13.4)
Unspecified	11
Modified NIH consensus criteria	
Very low risk	23 (7.8)
Low risk	120 (40.8)
Intermediate risk	74 (25.2)
High risk	77 (26.2)
Unspecified	1
Mutation analysis^a^	256 (86.8)
*KIT* exon 11	177 (69.1)
Substitution	63 (24.6)
Duplication/insertion	28 (10.9)
Deletion/insertion-deletion	86 (33.6)
Del557/558	47 (18.4)
Not del557/558	39 (15.2)
*KIT* exon 17	6 (2.3)
*PDGFRA* exon 12	9 (3.5)
*PDGFRA* exon 14	3 (1.2)
*PDGFRA* exon 18	50 (19.5)
D842V	40 (15.6)
Other	10 (3.9)
No *PDGFRA* or *KIT* mutation detected	11 (4.3)
SDHB expression positive	6
SDHB expression negative	2
Analysis not performed	3
Adjuvant imatinib	47 (15.9)
Duration, months [median (range)]	24 (1–60)
Neoadjuvant imatinib	11 (3.7)
Duration, months [median (range)]	8 (1–14)

Data are expressed as *n* (%) unless indicated otherwise

^a^Twenty-three patients in whom mutation analysis was not performed and 16 patients in whom tumour tissue was unfit for analysis were excluded

*GIST* gastrointestinal stromal tumor, *HPF* high-power field of the microscope, *NIH* National Institutes of Health, *PDGFRA* platelet-derived growth factor receptor-α, *SDHB* succinate dehydrogenase complex subunit B

**Table 2:** Associations between clinical, anatomical, and molecular characteristics

	Gastric subsite^a^				Growth pattern^b^				Genotype^c^			
	Upper	Middle	Lower	*p* value	Luminal	Exophytic	Transmural	*p* value	Del557/558	*KIT* not del557/558	*PDGFRA*	*p* value
Sex				0.089^f^				0.698				0.010
Male	70 (47.3)	52 (35.1)	26 (17.6)		49 (33.3)	45 (30.6)	53 (36.1)		31 (24.0)	60 (46.5)	38 (29.5)	
Female	52 (36.1)	68 (47.2)	24 (16.7)		51 (35.0)	49 (33.6)	46 (31.5)		16 (13.8)	76 (65.5)	24 (20.7)	
Tumor size, cm				0.214				< 0.001				< 0.001
≤ 10	103 (42.4)	95 (39.1)	45 (18.5)		99 (40.7)	71 (29.2)	73 (30.0)		28 (14.0)	123 (61.5)	49 (24.5)	
> 10	19 (38.8)	25 (51.0)	5 (10.2)		1 (2.0)	23 (46.0)	26 (52.0)		19 (42.2)	13 (28.9)	13 (28.9)	
Mitotic index				0.006				< 0.001				< 0.001
0–5	82 (37.4)	90 (41.1)	47 (21.5)		91 (41.6)	75 (34.2)	53 (24.2)		13 (7.4)	105 (60.0)	57 (32.6)	
6–10	11 (44.0)	12 (48.0)	2 (8.0)		7 (28.0)	9 (36.0)	9 (36.0)		2 (9.1)	19 (86.4)	1 (4.5)	
> 10	24 (64.9)	12 (32.4)	1 (2.7)		2 (5.3)	6 (15.8)	30 (78.9)		24 (64.9)	10 (27.0)	3 (8.1)	
Tumor rupture				0.092^g^				0.001				0.001
No	111 (41.1)	109 (40.3)	50 (18.5)		99 (36.7)	87 (32.2)	84 (31.1)		36 (16.1)	129 (57.8)	58 (26.0)	
Yes	11 (50.0)	11 (50.0)	0		1 (4.3)	7 (30.4)	15 (65.2)		11 (50.0)	7 (31.8)	4 (18.2)	
Risk category^d,e^				0.029				< 0.001				< 0.001
Non-high-risk	83 (38.2)	90 (41.5)	44 (20.3)		94 (43.5)	68 (31.5)	54 (25.0)		14 (8.1)	112 (64.7)	47 (27.2)	
High-risk	38 (51.3)	30 (40.5)	6 (8.1)		6 (7.9)	26 (34.2)	44 (57.9)		32 (45.1)	24 (33.8)	15 (21.1)	
Gastric subsite^a^								0.018				< 0.001
Upper	–	–	–		40 (32.8)	32 (26.2)	50 (41.0)		33 (30.0)	70 (63.6)	7 (6.4)	
Middle	–	–	–		35 (29.4)	45 (37.8)	39 (32.8)		13 (13.5)	50 (52.1)	33 (34.4)	
Lower	–	–	–		24 (48.0)	17 (34.0)	9 (18.0)		1 (2.6)	15 (39.4)	22 (57.9)	
Growth pattern^b^				0.018								< 0.001
Luminal	40 (40.4)	35 (35.4)	24 (24.2)		–	–	–		4 (5.0)	56 (70.0)	20 (25.0)	
Exophytic	32 (34.0)	45 (47.9)	17 (18.1)		–	–	–		9 (11.4)	44 (55.7)	26 (32.9)	
Transmural	50 (51.0)	39 (39.8)	9 (9.2)		–	–	–		34 (39.5)	36 (41.9)	16 (18.6)	
Genotype^c^				< 0.001				< 0.001				
Del557/558	33 (70.2)	13 (27.7)	1 (2.1)		4 (8.5)	9 (19.1)	34 (72.3)		–	–	–	
*KIT* not del557/558	70 (51.9)	50 (37.0)	15 (11.1)		56 (41.2)	44 (32.4)	36 (26.5)		–	–	–	
*PDGFRA*	7 (11.3)	33 (53.2)	22 (35.5)		20 (32.3)	26 (41.9)	16 (25.8)		–	–	–	

Data are expressed as *n* (%)

^a^Three patients with unspecified subsite were excluded

^b^Two patients with unspecified growth pattern were excluded

^c^Eleven patients with *KIT/PDGFRA* wild-type were excluded

^d^One patient at unspecified risk was excluded

^e^Modified National Institutes of Health criteria

^f^*p* = 0.053 when calculating upper versus middle/lower third

^g^*p* = 0.027 when calculating upper/middle versus lower third

*GIST* gastrointestinal stromal tumor, *PDGFRA* platelet-derived growth factor receptor-α

### Mutation Analysis

Mutation analysis was routinely performed on all intermediate- and high-risk tumors, and selectively on tumors at low or very low risk. Genomic DNA was extracted from fresh frozen or paraffin-embedded tumor tissue, and exons 9, 11, 13, and 17 of *KIT* and exons 12, 14, and 18 of *PDGFRA* were analyzed by Sanger sequencing and categorized as described previously.^17^ Tumors not analyzed routinely were analyzed using the AmpliSeq for Illumina Cancer Hotpot Panel version 2, examining hotspot regions of 50 cancer genes, including *KIT* exons 2, 9, 10, 11, 13, 15, 17, and 18, and *PDGFRA* exons 12, 14, 15, and 18. NGS libraries were generated from 100 ng of genomic DNA following Illumina’s protocol. Libraries were normalized using the AmpliSeq Library Equalizer kit from Illumina and sequenced paired end 2 × 150 base pairs on an Illumina MiSeq instrument. Sequence reads were mapped to the Human UCSC hg19 reference genome and variants called using the DNA Amplicon workflow version 2.1.0.19 using Illumina’s Local Run Manager software. Mutation calls were manually inspected on the Integrative Genomics Viewer.

### Statistical Analysis

RFS was estimated using the Kaplan–Meier method and compared using the log-rank test. Multivariable survival analysis was performed using the Cox proportional hazards regression model. Associations between variables were investigated using the two-tailed Fisher’s exact test or Pearson’s Chi-square test for categorical variables and independent Mann–Whitney *U* test or Kruskal–Wallis test for continuous variables. Interobserver agreement was evaluated using the Cohen’s *κ* test. A *p* value < 0.05 was considered statistically significant. IBM SPSS Statistics for Windows version 25.0 (IBM Corporation, Armonk, NY, USA) was used.

## Results

During the study interval, 297 patients underwent complete resection of primary gastric GIST. Two patients had multifocal gastric disease and were not included in the study cohort, which therefore comprised 295 patients. According to the mNIH criteria, 77 tumors (26.2%) were classified as high-risk (Table 1). Forty-seven patients (15.9%), all at high risk, received adjuvant imatinib. In 14 (29.8%) patients, treatment was prematurely discontinued. Eleven patients (3.7%) received neoadjuvant treatment; only one of these had a tumor ≤ 10 cm without rupture and was hence assigned to the unspecific risk category (Tables 1, 2). Treatment was continued postoperatively in 10 patients. Three patients were still being treated by the end of the study period. Further characteristics are shown in Table 1.

### Tumor Location

**Fig. 2:**
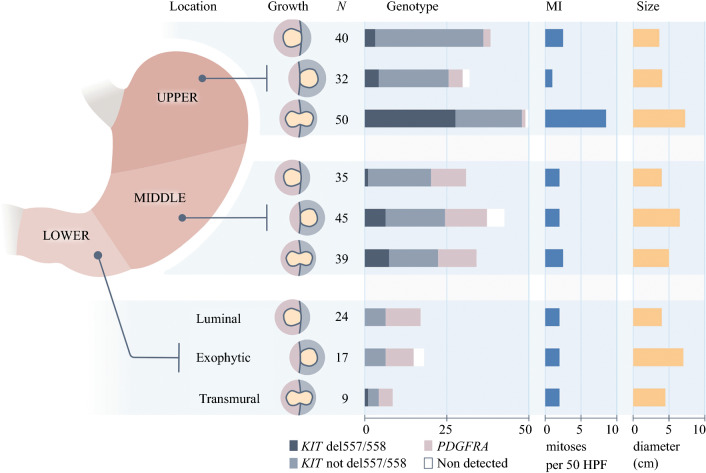
Relationships between tumor subsite, growth pattern, tumor size, mitotic index, and tumor genotype in gastric GIST. *GIST* gastrointestinal stromal tumor, *MI* mitotic index, *HPF* high-power fields of the microscope, *PDGFRA* platelet-derived growth factor receptor-*α*

Overall, 122 tumors were located in the upper third of the stomach (41.8%), 120 in the middle third (41.1%), and 50 in the lower third (17.1%) [Table 1 and Fig. 2]. There was no difference in size between tumors at different sites (Fig. 2 and electronic supplementary Fig. S1a). Mitotic activity was higher in tumors of the upper third, with a median MI of 3 (range 0–130) versus 2 (range 0–166) for those in the middle third, and 2 (range 0–12) for those in the lower third (*p *= 0.020) [electronic supplementary Fig. S1d). Non-high-risk tumors were more common in the lower third compared with the upper two thirds: 88.0 versus 71.8% (*p *= 0.019) (Table 2).

### Tumor Growth Pattern

The distribution of luminal, exophytic, and transmural tumors was even (Table 1). The proportion of transmural tumors was higher in the upper third (41.0% vs. 28.4% in the middle/lower third; *p *= 0.032) (Table 2 and Fig. 2). Transmural tumors had a higher MI (median 3 [range 0–166]) than exophytic and luminal tumors (median 2 [range 0–32] and 2 [range 0–14], respectively; *p *< 0.001 (electronic supplementary Fig. S1e). Transmural tumors were also larger (median size 6.0 cm [range 2.0–30.0]) than exophytic (median size 5.3 cm [range 0.5–34.0]) and luminal tumors (median size 4.0 cm [range 0.5–10.2]; *p *< 0.001) [electronic supplementary Fig. S1b]. Of ruptured tumors, 15 were transmural, 7 were exophytic, and 1 was luminal (*p *= 0.001). Forty-five per cent of transmural tumors were high-risk, compared with 16.5% of exophytic/luminal tumors (*p *< 0.001).

### Tumor Genotype

Mutation analysis was successfully performed in 256 patients (86.8%) [Table 1]. Only 11 (4.3%) patients had tumors wild-type for *PDGFRA/KIT*. The majority of *PDGFRA*-mutated tumors were located in the lower two-thirds of the stomach, whereas *KIT*-mutated tumors had a predilection for the upper third (Table 2 and Fig. 2). This pattern was even more pronounced for del557/558-mutated tumors: 33 (70.2%) were located in the upper third and only one (2.1%) was located in the lower third. In the upper third, only 9 of 113 patients (8.0%) had tumors with genotypes insensitive to imatinib (*PDGFRA* exon 18 D842V or *PDGFRA*/*KIT* wild-type). *PDGFRA* and other than del557/558 *KIT*-mutated tumors typically presented a luminal or exophytic pattern, whereas 72.3% of tumors with a del557/558 mutation were transmural (*p *< 0.001) [Table 2]. Del557/558-mutated tumors had a higher mitotic activity than tumors with other *KIT* and *PDGFRA* mutations (median 12 [range 0–166] vs. median 2 [range 0–53]; *p *< 0.001) [Table 2 and electronic supplementary Fig. S1f]. Rupture was recorded in 11 tumors (23.4%) with del557/558, and in 12 tumors (5.7%) of other genotypes (*p *= 0.001). Among 134 males with known mutation status, 31 had del557/558-mutated tumors (23.1%), whereas this genotype was detected in only 17 of 122 females (13.9%) [*p* = 0.037].

### Recurrence

**Fig. 3:**
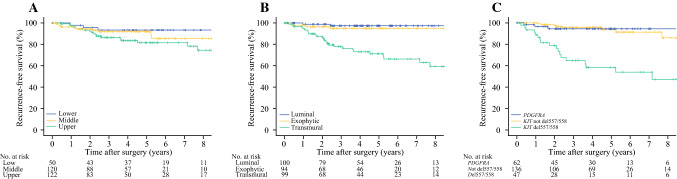
Estimated recurrence-free survival (Kaplan–Meier) after resection of gastric GIST by **a** gastric subsite (*p* = 0.020); **b** macroscopic growth pattern (*p* < 0.001); and **c** tumor genotype (*p* < 0.001). *GIST* gastrointestinal stromal tumor, *PDGFRA* platelet-derived growth factor receptor-*α*

All patients were included in the survival analysis. After a median follow-up period of 46 months (range 0–185), 35 recurrences were documented. Estimated 5-year RFS was 88%. RFS was shorter for patients with GIST in the upper third than patients with GIST in the middle or lower thirds (81% vs. 92% at 5 years; hazard ratio (HR) 2.25, 95% confidence interval [CI] 1.12–4.52; *p* = 0.020) [Fig. 3a]. Only seven recurrences were seen in patients with luminal or exophytic tumors, corresponding to a 5-year RFS of 96%. With transmural tumors, 5-year RFS was 71% (HR 8.45, 95% CI 3.69–19.36; *p* < 0.001) [Fig. 3b]. In the high-risk category, 5-year RFS was 46% with transmural tumors versus 83% with luminal or exophytic tumors (HR 4.47, 95% CI 1.71–11.66; *p* = 0.001). Patients with del557/558-mutated tumors had a 5-year RFS of 58%, which was inferior to patients with other than del557/558 *KIT* mutations, i.e. 94% (HR 8.43, 95% CI 3.71–19.17; *p* < 0.001) and patients with *PDGFRA* mutations, i.e. 95% (HR 9.65, 95% CI 2.86–32.59; *p* < 0.001) [Fig. 3c]. With tumor rupture, 5-year RFS was 36% versus 93% without rupture (HR 15.29, 95% CI 7.78–30.01; *p* < 0.001. In multivariable survival analysis with sex, tumor size, MI, tumor rupture, growth pattern, and genotype as covariates, only MI and tumor rupture were independently related to recurrence (HR 22.23, 95% CI 5.06–97.60, *p* < 0.001; and HR 7.53, 95% CI 1.50–37.76, respectively, *p* = 0.014) [electronic supplementary Table S1].

### Tumor Growth Pattern as a Supplement to Established Risk Stratification Criteria

When information on mitotic activity is missing, e.g. after neoadjuvant therapy, tumor size and rupture are the only established criteria for risk stratification. However, transmural growth was strongly associated with high MI, and the results reported above indicate that patients with luminal or exophytic tumors without rupture have a good prognosis despite an unfavorable tumor size. We therefore investigated patients with tumors larger than 5 cm. This group included 60 patients with transmural tumors (44.8%), 73 patients with exophytic or luminal tumors (54.5%), and one patient with unspecified tumor growth pattern. Among the 73 patients with exophytic or luminal tumors, 7 patients had tumor rupture; only five recurrences were observed, four of which had tumor rupture. In the 66 patients with exophytic or luminal tumors > 5 cm without rupture, 5-year RFS was 98%. Fifty-five of these 66 patients (83.3%) did not receive adjuvant treatment. By contrast, 45 patients had transmural tumors > 5 cm without rupture, among which 10 recurrences were detected, corresponding to an estimated 5-year RFS of 77% (*p* < 0.001). In this group, 26 patients (57.8%) did not receive adjuvant treatment.

## Discussion

The present investigation has documented that tumor genotypes have a characteristic anatomical distribution in gastric GIST. This finding is clinically relevant, as over 90% of tumors in the upper third harbored mutations sensitive to imatinib. Furthermore, a transmural growth pattern was strongly associated with high mitotic activity and recurrent disease. By contrast, patients with exophytic or luminal tumors rarely relapsed, even when assigned to the high-risk category.

A rare disease, and still a young entity, much of our understanding of GIST has been provided by academic centers with biased referral patterns or trials with selected participants. In these reports, the proportion of tumors wild-type for *KIT/PDGFRA* is stated to be 10–20%, higher than the 4% in the present series.^9^,^10^ This discrepancy may also reflect technical shortcomings in old studies. *PDGFRA*-mutated tumors had a strong predilection for the middle and lower third of the stomach. In the upper third, 92% of the tumors harbored genotypes sensitive for imatinib, and neoadjuvant treatment might be started without mutation analysis. In countries and centers lacking facilities for molecular testing, adjuvant treatment could also be considered for high-risk tumors in this location.

For patients with GIST in the upper end of the stomach, RFS was shorter than with tumors in the lower end. This observation was made by Miettinen and colleagues in 2005, but has been given little attention.^18^ In the current study, among patients with tumors in the lower third, there were only three recurrences, representing an estimated 5-year RFS of 93%, versus 81% for patients with tumors in the upper third. This difference seems related to mitotic activity and the distribution of tumor genotypes, more specifically to tumors with a del557/558 mutation. The association between del557/558-mutated tumors and an elevated MI has been described by others,^9^ but their concurrence in the upper part of the stomach, as well as the overrepresentation of del557/558-mutated tumors in gastric GISTs of men, are, to our knowledge, novel findings.

GISTs originate from the interstitial cells of Cajal, situated at different levels of the gastric wall. *A priori,* their direction of growth would be decided by the depth of the proliferating cells and the tenacity of the surrounding tissue, and unrelated to inherent biological factors. Nevertheless, there were associations between growth patterns and risk factors that suggest a more complex explanation: transmural tumors had both a higher MI and a higher frequency of del557/558 mutations than luminal or exophytic tumors. Most importantly, patients with transmural tumors had significantly reduced RFS. This difference remained when tumors of the low-risk lower third were excluded (*p* < 0.001), when ruptured tumors were excluded (*p* < 0.001), when del557/558-mutated tumors were excluded (*p* < 0.001), and when tumors > 10 cm were excluded (*p* < 0.001). However, there was a strong association between a transmural growth pattern and high mitotic activity. Neoadjuvant treatment with imatinib is increasing and precludes the assessment of MI. In these patients, risk stratification is uncertain, often impossible. When information on mitoses is missing, our data suggest that growth pattern can be used as a surrogate variable after neoadjuvant therapy. Such treatment is rarely indicated for tumors smaller than 5 cm, but in larger tumors without rupture, withholding continued adjuvant treatment for those with exophytic or luminal tumors seems safe. In the present cohort, this group would have comprised 66 patients, including 22 truly at high risk, and there was only one recurrence. A minority of these patients (17%) received adjuvant treatment.

Del557/558 mutations were closely associated with established risk factors of greater intrinsic impact, and recommending adjuvant imatinib for patients with del557/558-mutated tumors outside the high-risk category, as proposed by some authors,^9^ is not supported by the present data.

This study has some limitations, most importantly its retrospective design and the inconsistent use of adjuvant imatinib, which confounds the analysis of recurrence. Of 35 patients who relapsed, 20 received adjuvant therapy. However, among the 15 patients who did not receive adjuvant therapy, 2 patients had low-risk tumors and would not have been treated by any standard, 3 patients at high risk had imatinib-insensitive tumors, and, of the remaining 10 patients, 6 had high-risk transmural tumors with imatinib-sensitive genotypes and would have received treatment according to our suggested recommendations.

## Conclusions

In this population-based study of patients with gastric GIST, tumor genotypes had a characteristic anatomical distribution. In the upper third, over 90% of tumors were sensitive to imatinib. A transmural growth pattern was a predictor of poor outcome, related to an elevated MI. Exophytic and luminal tumors, irrespective of size, were associated with an excellent prognosis without rupture, and these patients may not benefit from adjuvant treatment.

## Supplementary Information

Below is the link to the electronic supplementary material.

Supplementary material 1 (DOCX 18 kb)

Supplementary material 2 (TIF 624 kb)
