# Supplementary material for: Integrating Anatomical, Molecular and Clinical Risk Factors in Gastrointestinal Stromal Tumor of the Stomach
Source: Ann Surg Oncol. 2021 Mar 2;28(11):6837–45. doi: 10.1245/s10434-021-09605-8 (PMC8460510; doi:10.1245/s10434-021-09605-8)
Supplement: Supplementary file 1 — Supplementary material 1 (DOCX 18 kb) [file 10434_2021_9605_MOESM1_ESM.docx]

Supplementary Table S1. Multivariable Cox regression analysis of recurrence-free survival

|  | HR | 95% CI | *P* value |
| --- | --- | --- | --- |
| Sex |  |  | 0∙922 |
| Female | 1∙00 (reference) |  |  |
| Male | 1∙05 | 0∙40-2∙75 |  |
| Tumor size |  |  | 0∙278 |
| ≤ 10 cm | 1∙00 (reference) |  |  |
| > 10 cm | 0∙40 | 0∙08-2∙10 |  |
| Mitotic index |  |  | < 0∙001 |
| ≤ 10 per 50 HPF | 1∙00 (reference) |  |  |
| > 10 per 50 HPF | 22∙23 | 5∙06-97∙60 |  |
| Tumor rupture |  |  | 0∙014 |
| No | 1∙00 (reference) |  |  |
| Yes | 7∙53 | 1∙50-37∙76 |  |
| Tumor growth pattern |  |  | 0∙120 |
| Luminal or exophytic | 1∙00 (reference) |  |  |
| Transmural | 2∙90 | 0∙76-11∙06 |  |
| Tumor genotype |  |  | 0∙349 |
| *PDGFRA* | 1∙00 (reference) |  |  |
| *KIT* not del557/558 | 0∙41 | 0∙09-1∙82 |  |
| *KIT* del557/558 | 0∙30 | 0∙06-1∙54 |  |

HR, hazard ratio; CI, confidence interval; HPF, high-power field of the microscope; *PDGFRA*, platelet-derived growth factor receptor-α.
